# Polyostotic Fibrous Dysplasia: A Case Report of Rarity

**DOI:** 10.7759/cureus.36403

**Published:** 2023-03-20

**Authors:** Sanjana N Wadewale, Nitin D Bhola, Anchal Agarwal

**Affiliations:** 1 Oral and Maxillofacial Surgery, Sharad Pawar Dental College and Hospital, Datta Meghe Institute of Medical Sciences, Wardha, IND

**Keywords:** maxilla, ground glass, polyostotic, fibrous dysplasia, craniofacial

## Abstract

A skeletal condition known as fibrous dysplasia (FD) is characterized by the replacement of healthy bone with fibrous bone tissue. One bone (monostotic) or several bones could be involved (polyostotic). Any bone in the body might become affected by FD. The skull and face bones are the most typical locations. It is connected to a GNAS1 gene mutation (20q13.2). It begins during childhood and could continue far into adolescence and adulthood. In this case study, a 22-year-old woman was identified as having polyostotic FD based on her clinical, radiological, and histological characteristics.

## Introduction

Lichtenstein described fibrous dysplasia (FD) in the year 1938 [1]. The incidence rate of FD is 1:4000-1:10,000 [2]. It seems to be a rare disease. In the craniofacial region, it is more common in the upper jaw compared to the lower jaw [3]. FD is "a benign lesion, presumably developmental in nature, characterized by the presence of fibrous connective tissue with a characteristic whorled pattern and containing trabeculae of immature non-lamellar bone", Waldron 1985 [4]. An active mutation in the gene that codes for the subunit of stimulatory G protein, which is situated at "20q13.2-13.3", has been connected to the etiology of FD [5]. It accounts for 7% of benign bone tumors and 2.5% of all bone abnormalities. These illnesses disproportionately affect men and boys [3]. One bone (monostotic type) or several bones may be affected (polyostotic type). The latter may form part of the McCune-Albright syndrome (MAS) or the Jaffe-Lichtenstein syndrome (JLS). The difference between MAS and JLS is they include other endocrinopathies due to the hyperactivity of various endocrine glands [6]. This case of polyostotic FD in a 22-year-old woman who presented to the outpatient Department of Oral and Maxillofacial Surgery is being reported.

## Case presentation

A 22-year-old female presented with painless swelling over the right side of her face for the past seven years. When the patient first observed swelling on the right side of her face, it had no prior history of prodromal symptoms and was slowly growing and progressing. The patient had a previous history of gross facial asymmetry and nasal congestion for four to five years approx. The patient has no history of trauma or other associated diseases related to FD.

**Figure 1:**
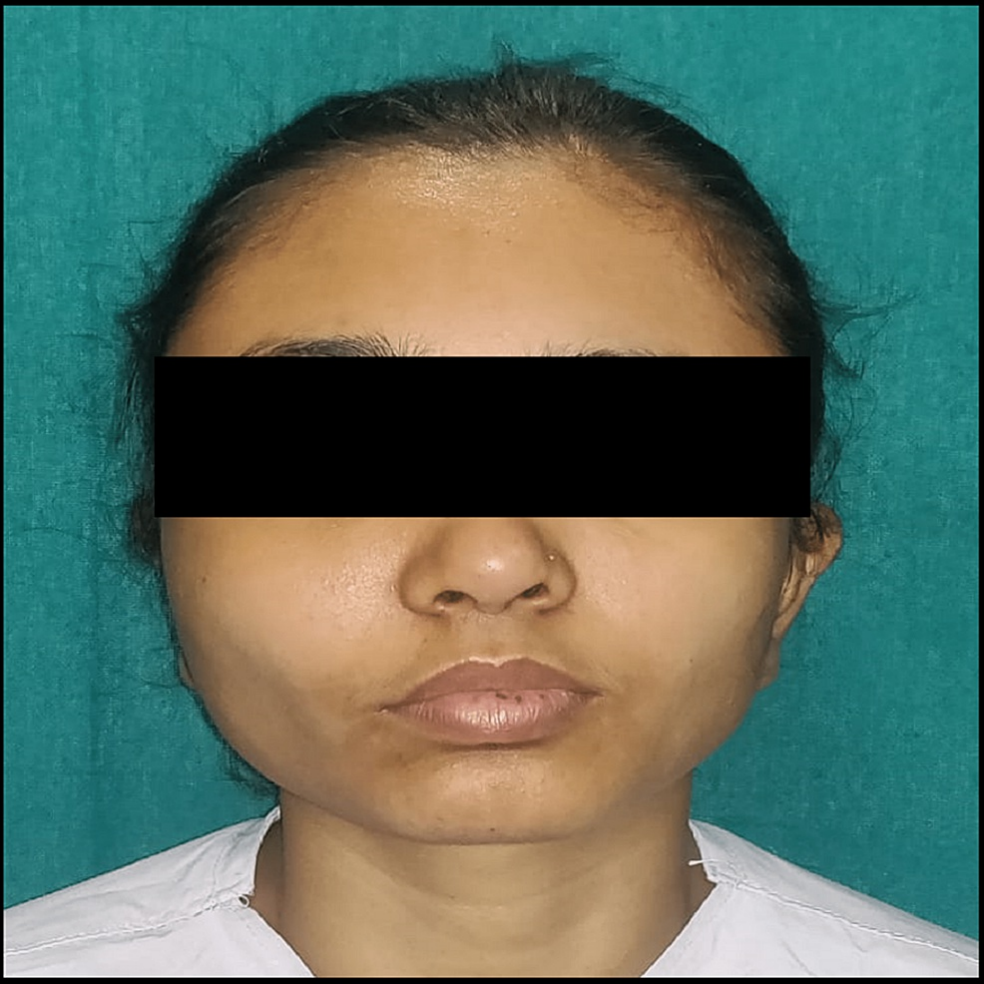
Clinical photograph showing unilateral swelling over the right side of the face

The right side of the face had a well-defined, hard, bony swelling, frontal bossing, loss of the right nasolabial fold, and increased inter-canthal and interpupillary distance upon extraoral examination (Figure 1).

**Figure 2:**
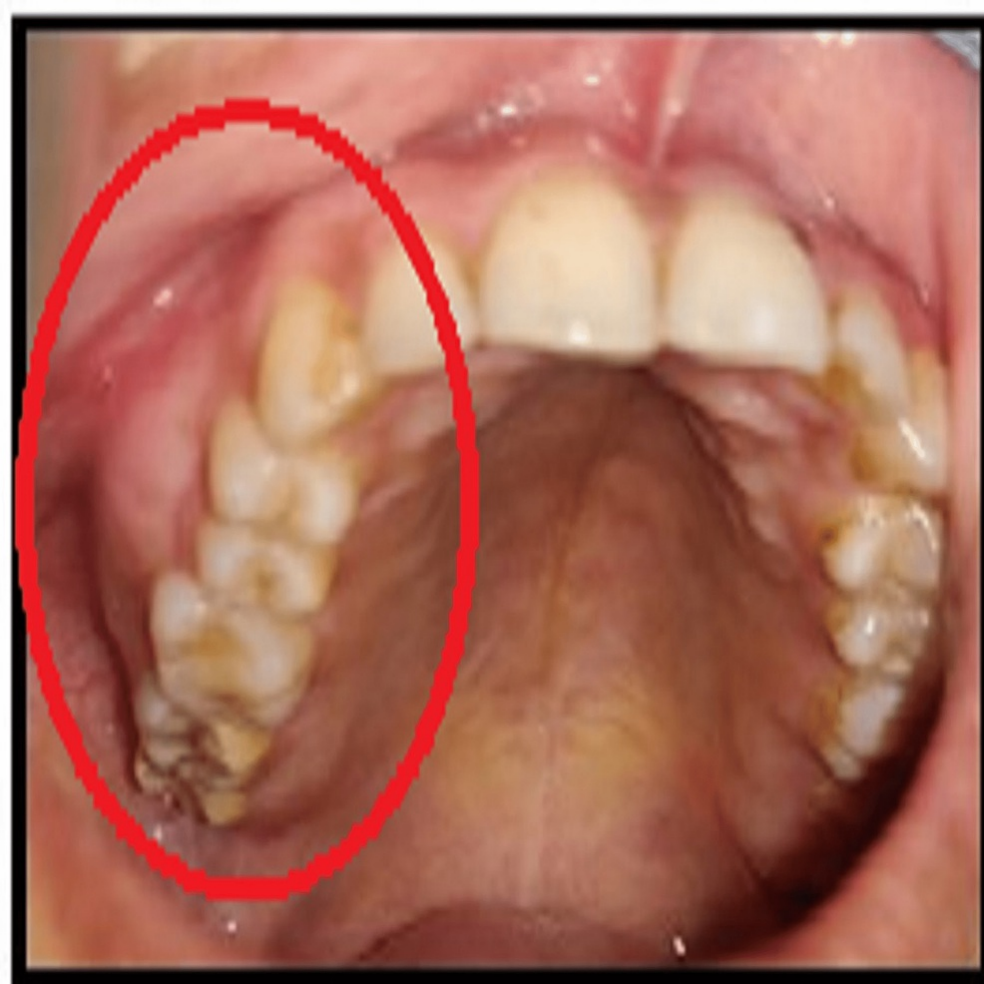
Intraoral photograph showing the expansion of buccal cortex over the right side of the maxilla

Cafe-au-lait skin spots with irregular borders on the dorsal surface of the body were present. Intraorally there was expansion of buccal cortex of right posterior region of maxilla and obliteration of right buccal maxillary vestibule (Figure 2).

**Figure 3:**
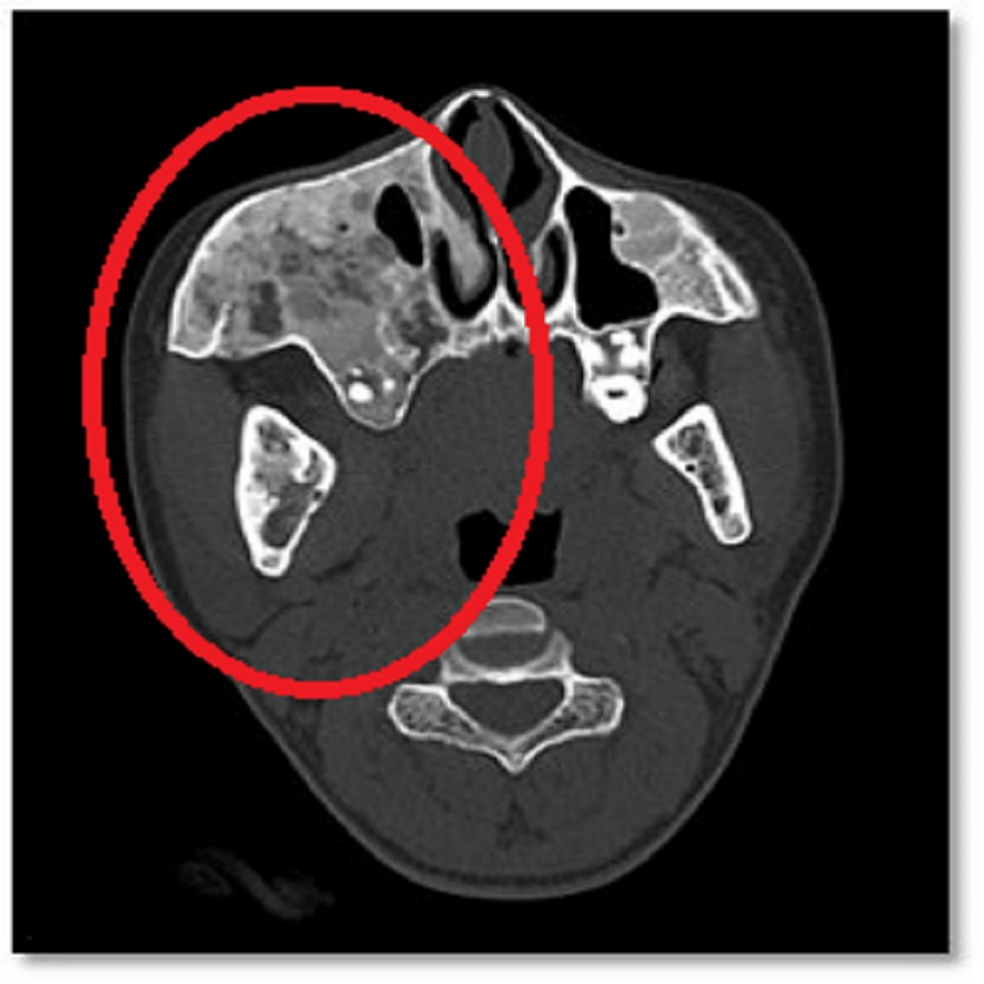
CECT head (axial cut) showing diffuse ill-defined expansile ground glass lesion involving craniofacial region CECT: contrast-enhanced computed tomography

**Figure 4:**
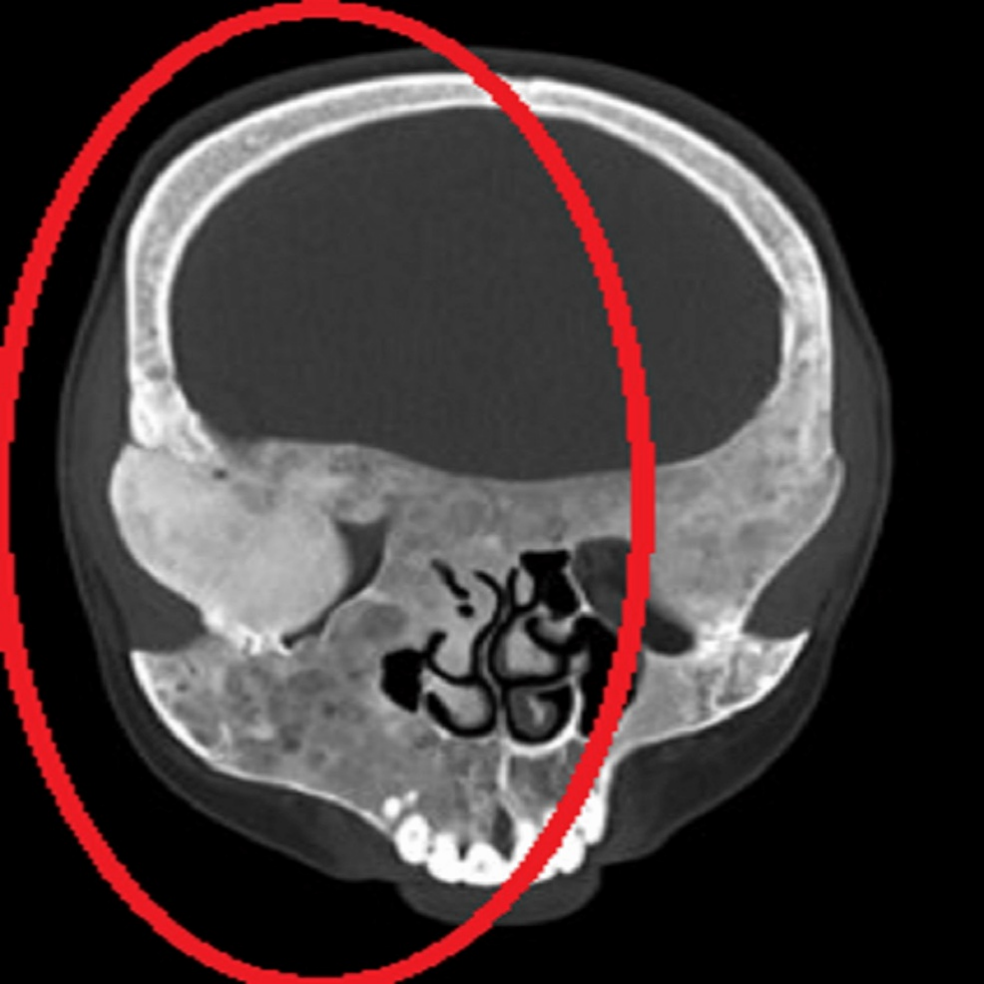
CECT head (coronal cut) showing diffuse ill-defined expansile ground glass lesion involving craniofacial lesion CECT: contrast-enhanced computed tomography

**Figure 5:**
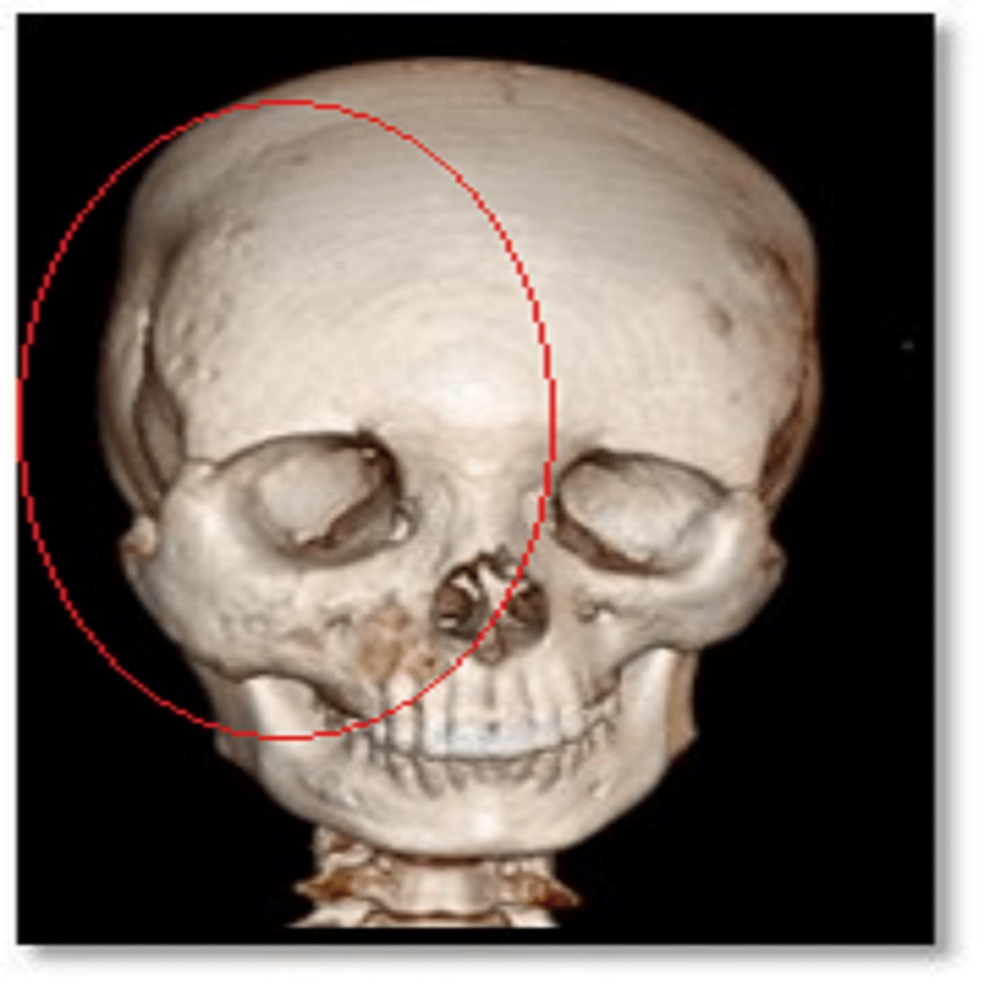
CECT head (3D cut) showing bony expansion over the right craniofacial region CECT: contrast-enhanced computed tomography

Based on clinical examination, the provisional diagnosis was a fibro-osseous lesion. To confirm our diagnosis, we did contrast-enhanced computed tomography (CECT) of the head and neck, an X-ray of the lower limb, a routine blood investigation, and a parathyroid hormone test. CECT of the head and neck revealed expansion with ground glass matrix involving the medullary cavity. There was the involvement of bilateral temporal, occipital, parietal, and frontal bones, bilateral turbinates sparing the left inferior turbinate, lamina papyracea, walls of bilateral orbits, bilateral sphenoid wings, and pterygoid plates-evidence of narrowing right maxillary sinus and nasal cavity (Figures 3-5).

**Figure 6:**
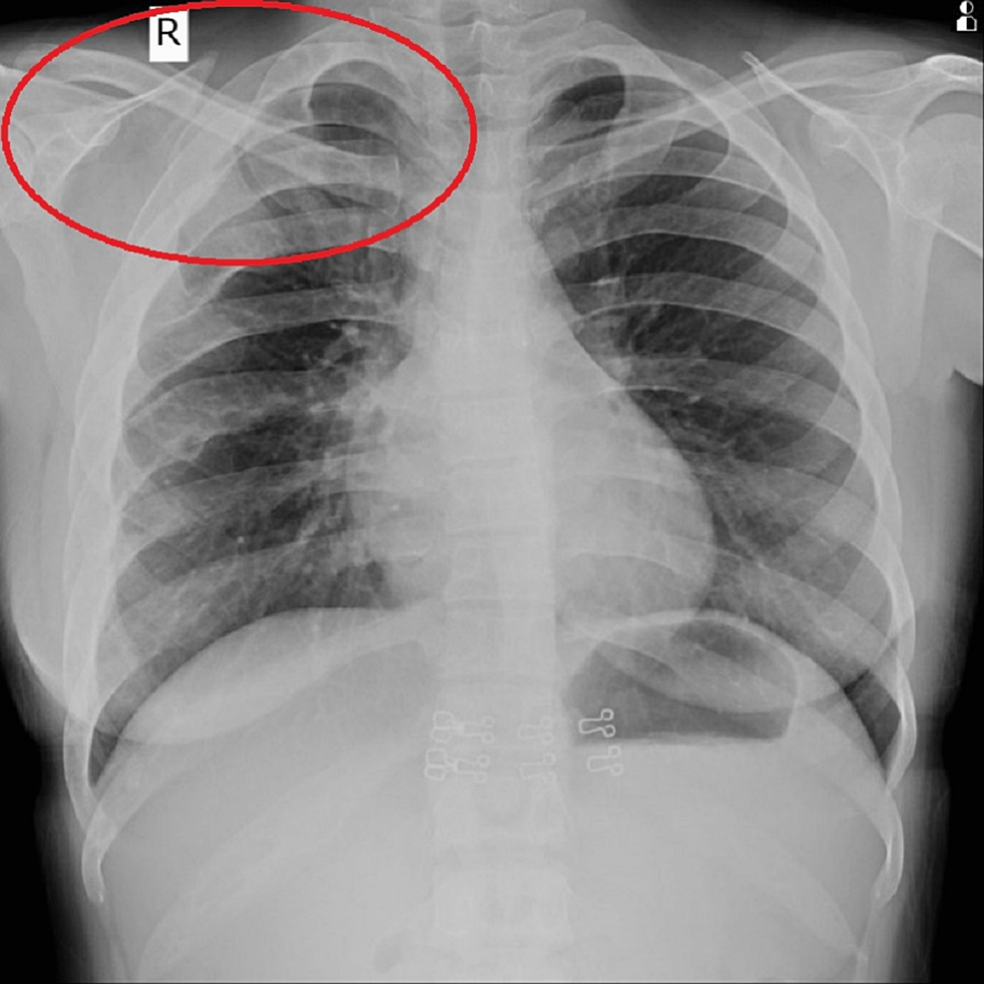
Chest X-ray showing the expansion of right scapula, first and second rib, and sternum

The bony calvarium was widening, relatively sparing the left hemimandible. The ramus, body, and condyle of the right hemimandible and right maxilla were involved. The odontoid process, bilateral lamina and body of the C2 vertebra, and right lamina of the C3 vertebra are also involved. Similar changes were noted in visualized part of the sternum, first and second ribs on the right side, and right scapula (Figure 6).

**Figure 7:**
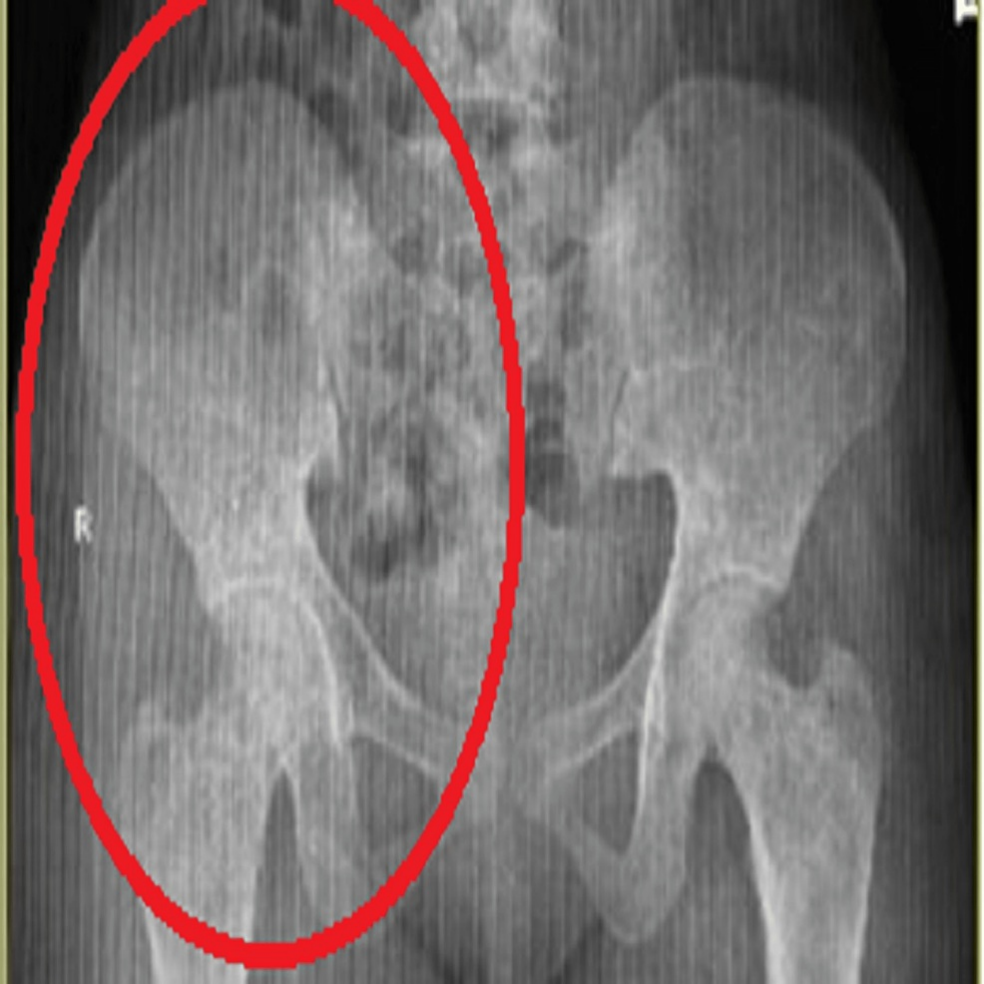
X-ray of pelvis showing lytic sclerosis

X-ray of the femur, humerus, and pelvis revealed a short alternate area of lytic sclerosis (Figure 7).

High serum alkaline phosphatase (ALP) (325 IU/L) and parathyroid hormone (64.5 pmol/L) in blood, low luteinizing hormone (3.45 IU/L) and vitamin D (13.8 ng/mL) in blood. Clinical, radiological, and hematological findings brought us to the final diagnosis of polyostotic FD, specifically MAS.

**Figure 8:**
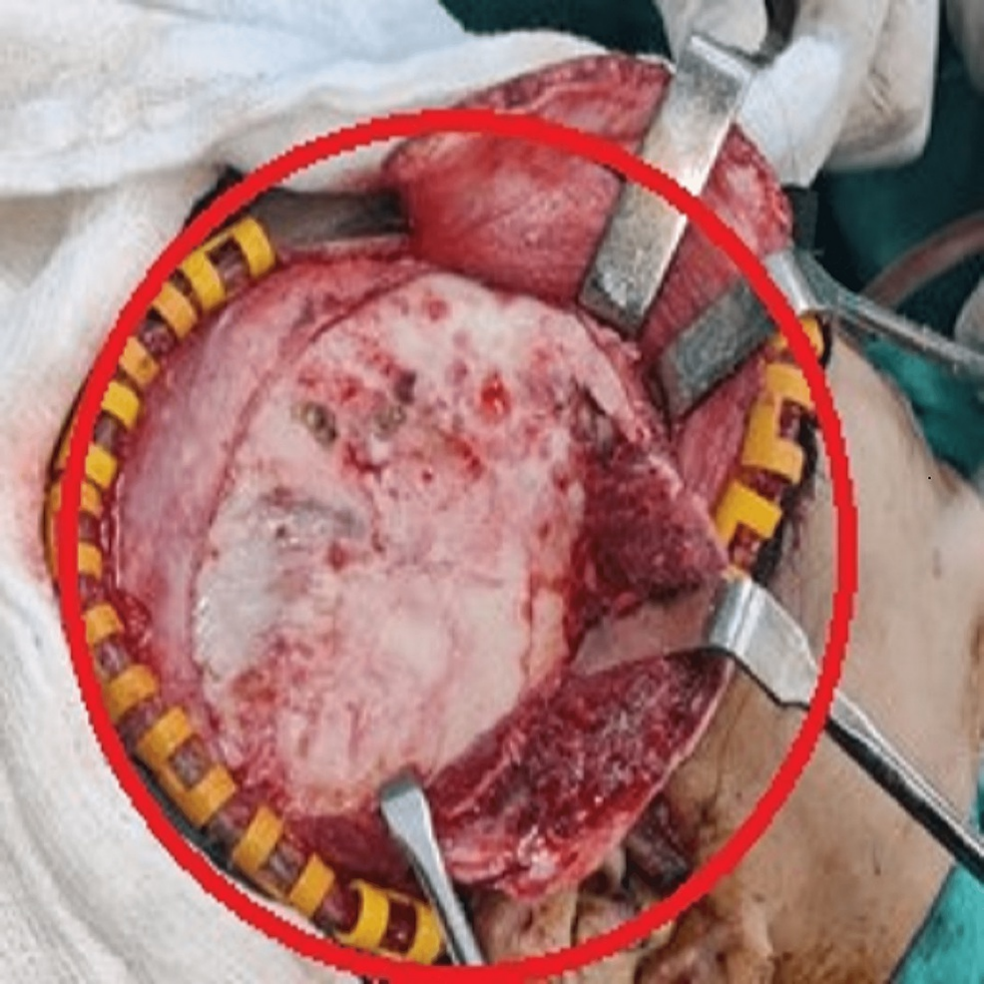
Photograph showing exposed recontoured frontal and temporal region of the right side

The treatment plan was conveyed to the patient, and consent was taken for surgical recounting. The normal side of the face was taken as a reference. Hemi-coronal incision extending up to the preauricular region given. The flap was raised, excess bone shaved, and the right frontal and temporal bone recontouring was done (Figure 8).

**Figure 9:**
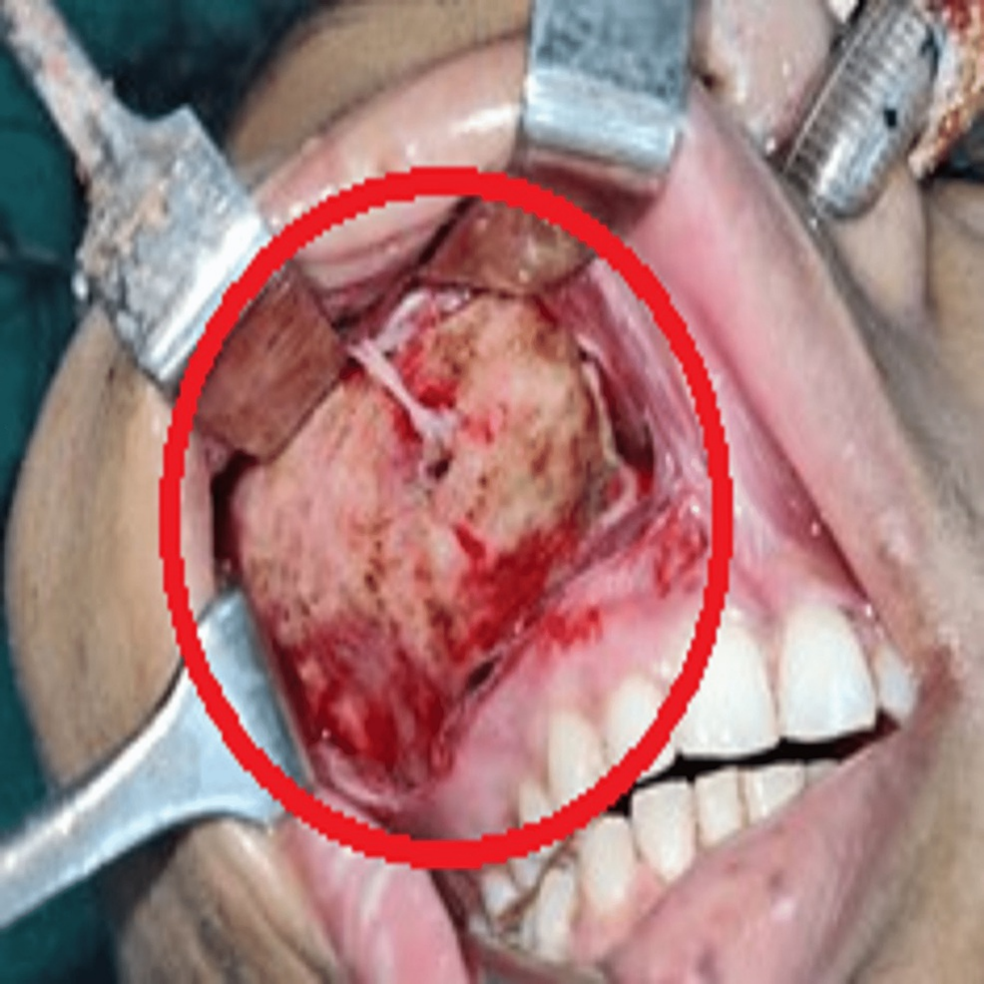
Photograph showing exposed recontoured of the right infra-orbital, zygomatic and maxillary region

The maxillary vestibular incision was given from the right upper incisor to the upper first molar of the right side, the flap was raised, and excess bone shaving and recounting were done of the right infra-orbital, zygomatic bone, and maxilla (Figure 9).

**Figure 10:**
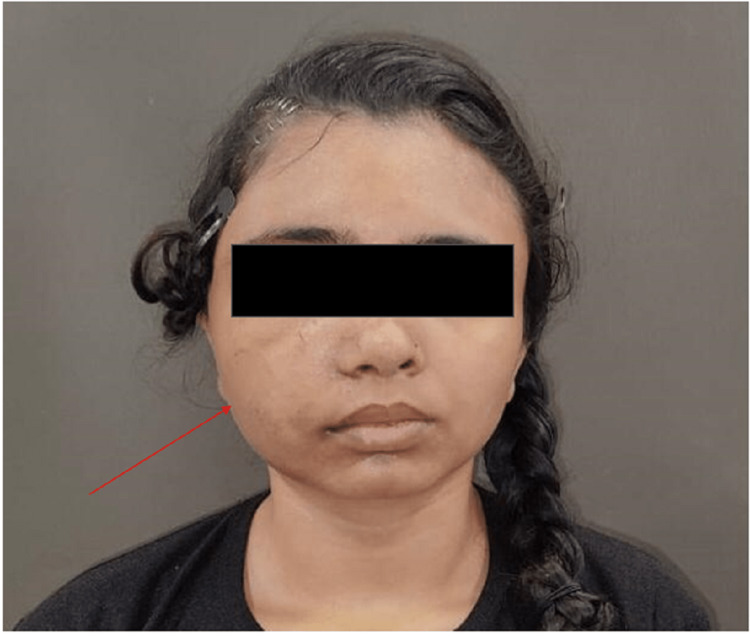
Showing a post-operative photograph of a patient

A histological examination of the recontoured bone revealed highly cellular connective tissue and bony trabeculae in the shape of Chinese letters. The presence of osteoblasts' peripherally and the presence of osteocytes in the trabeculae suggested FD. There have been no surgical problems while the patient is receiving standard follow-up (Figure 10).

## Discussion

FD is a reasonably frequent, benign condition that differs from other fibro-osseous lesions [7]. Before the biopsy or surgical specimen is available, a diagnosis of craniomaxillofacial fibrous dysplasia (CFD) can be made with considerable accuracy based on the patient's medical history and radiographic features. More frequently has an impact on the maxilla than the mandible [3]. The frontal, temporal, parietal, and maxillary regions are, nonetheless, involved in our case. Patients may express concerns about facial deformities, visual changes, nasal congestion, discomfort, or hearing impairment.

Our patient reported a non-painful facial swelling on the affected side. The anterior area is the least affected, and most tumors start in the premolar region and spread to the third molar region [8]. The same findings were seen in our case. Warning indications of cancerous transformation in FD have elevated levels of ALP [9]. As a result, its levels in these patients should be routinely checked. As a result of the patient's elevated ALP levels, an evaluation of her ALP should be done every six months [10]. Surgery for CFD usually seeks to rectify the facial deformities and restore the missing foramina when they lead to complications such as proptosis, orbital dystopia, nasal dysfunction, etc. [11].

There are several treatments, including observation, medication, surgery, and aggressive excision and reconstruction. The best course of treatment for minor, asymptomatic abnormalities of the craniomaxillofacial bone that the patient finds acceptable in terms of appearance is monitoring. To date, medical therapy has not played a significant part in the treatment of FD. Treatment with bisphosphonates was made possible by understanding the disease's pathophysiology. They prevent osteoclastic activity from limiting bone degradation. They can't easily break their association with the hydroxyapatite in the resorbed bone because of their tremendous affinity for it. The drug penetrates the cell's cytoplasm, suppressing the synthesis of acid phosphatase and preventing bone resorption. According to this notion, the medication stabilizes the condition and lessens pain for the patient [12]. Another drug mentioned in the literature is calcitonin. Vitamin D and calcium supplements have also been suggested because many patients have low serum calcium levels [13].

Even though there are no universally recognized standards for treating CFD, surgical therapy is still the backbone of care [14]. It aims to restore normal facial aesthetics and repair or prevent functional deficiencies. A remodeling process used in conservative surgery is intended to produce aesthetically attractive results [15]. However, this has a 15-20% chance of recurrence, particularly throughout the growth phase [2]. A more individualized approach is desirable in these circumstances, and the decision to treat is based on a thorough examination of the patient's disease from a functional and aesthetic perspective. The craniofacial region is unique since it is challenging to eradicate pathology there due to the proximity of important tissues. Treatment for CFD focuses on reaching aesthetic or practical objectives [16]. Typically, surgery is put off until puberty in the hopes that the condition will go into remission. Even though a cautious surgical resection is preferable, a more forceful approach may be necessary if the foramina and orbital apex are compressing the skull base. It is crucial to maintain a close eye on patients after conservative treatment.

For the management of CFD, "Chen and Noordhoff" presented a treatment algorithm in 1990 that included aggressive, radical surgery for the excision of diseased tissue. Based on the aesthetic and functional effects of the disease at each of these sites and the particular anatomic considerations for operating in each area, they suggested that the head and face may be separated into four zones for this algorithm. "The fronto-orbito-malar areas of the face are represented by Zone 1. These are crucial from an aesthetic standpoint and can be successfully rebuilt using basic bone grafting methods following repair. They advised complete excision and rebuilding for this area. The scalp with hair is referred to as Zone 2. The patient can choose not to intervene because it is often not an aesthetic concern. The central skull base, which includes the sphenoid, pterygoid, petrous temporal bone, and mastoid, is Zone 3. The authors advised monitoring lesions in this area because it was challenging to gain surgical access to these locations. The mandible, maxilla, and skull parts that support teeth make up Zone 4. Given the difficulty in reconstructing flaws in this region, the authors advised using a conservative management approach" [17].

According to estimates, FD and MAS both carry a 0.4% and 4% risk of malignant transformation, respectively, with the craniofacial region being the most common site of occurrence in both conditions [18]. Serum ALP levels are crucial for spotting recurrences of FD. After complete resection, the postoperative serum ALP level does not rise. Partial resection is recommended if the patient is an adult and has a condition that prevents total resection because the likelihood of recurrence is low [19]. No further therapy is necessary for adult patients who wish to have aesthetic surgery, regardless of their preoperative serum ALP levels. Surgical recontouring has a higher success rate than the usual approach, and although it is a demanding process, it is a permanent treatment that produces good functional and aesthetic outcomes [20].

## Conclusions

A rare benign bone tumor, FD most frequently affects the jaws. The surgeon should be able to distinguish from other bony lesions in the first place. Until the growth spurt is over, surgery should be postponed. As a result, the patient should be monitored throughout the procedure and on an ongoing basis. The most common type of treatment favored is conservative, such as shaping and recontouring. Another surgical therapeutic option is radical excision and reconstruction.
